# Comparing Bayesian random coefficient prediction and latent interaction models for multilevel moderated mediation

**DOI:** 10.3389/fpsyg.2025.1543330

**Published:** 2026-03-05

**Authors:** Sooyong Lee, Soyoung Kim

**Affiliations:** 1 Department of Education, University of Wisconsin-Madison, Madison, WI, United States; 2 Department of Psychology, Chonnam National University, Gwangju, Republic of Korea

**Keywords:** multilevel structural equation modeling, Bayesian estimation, mediation, cross-level moderation, mediated moderation

## Abstract

**Introduction:**

This study compares Bayesian random coefficient prediction (BRCP) and Bayesian latent interaction (BINT) models to detect moderated mediation effects in multilevel contexts.

**Materials and methods:**

We evaluated the performance of these models under various conditions using empirical data from the Trends in International Mathematics and Science Study (TIMSS2019) dataset and simulated data.

**Results:**

The results showed that the BRCP and BINT models produced highly similar parameter estimates with negligible differences.

**Discussion:**

The empirical findings revealed consistent within- and between-level relationships across both models, while simulation results indicated acceptable bias, controlled Type I error rates, and sufficient power in most conditions, except for smaller cluster sizes. We observed a slightly higher bias for BINT under small sample conditions. Overall, both models are effective for moderated mediation analysis, though BRCP is slightly more suitable for smaller samples.

**Conclusion:**

These findings highlight the robustness of Bayesian approaches in handling complex hierarchical data, particularly in educational and psychological research. Future research should explore additional factors, such as measurement error and more complex moderator structures, to enhance our understanding of Bayesian multilevel modeling.

## Introduction

In social and behavioral research, understanding the roles of mediation and moderation is essential for explaining relationships among variables. Mediation refers to the process through which an independent variable influences a dependent variable via a mediator, while moderation examines how the strength or direction of this relationship changes depending on the level of a moderator. The combination of these two concepts, known as moderated mediation, is also a useful analytical approach for exploring how the mediation process varies depending on the level of a third variable acting as a moderator (Muller et al., 2005). Scholars have frequently analyzed applications of moderated mediation without considering latent variables. Researchers widely use the regression-based approach (Hayes, 2017) because it does not require extensive knowledge of structural equation modeling (SEM), and it is approachable.

We can improve these practices by incorporating latent variables into moderated mediation analyses, thereby accounting for measurement errors and leading to more precise estimates of relationships. Moreover, multilevel data structures embed many research questions involving moderation, mediation, and moderated mediation. In fields such as education and psychology, data often has a hierarchical structure, such as students nested within schools or employees nested within organizations. These nested structures require hierarchical linear models (HLM) or multilevel modeling (MLM) techniques. To address these complexities, multilevel structural equation modeling (MSEM) offers a framework for testing complex structural relationships while considering the multilevel nature of data (Stapleton, 2013).

The MSEM framework provides two primary approaches for testing multilevel moderated mediation models: random coefficient prediction (RCP) and interaction-based models, initially proposed as latent moderated structural equations (LMS) models under a maximum likelihood estimation framework^1^ (Preacher et al., 2016). The RCP approach treats random slopes as latent variables at the between level, with moderation effects tested based on how these slopes vary according to covariates at higher levels. In contrast, the LMS framework incorporates interaction terms directly into the structural equation model, testing the moderation effect by including product terms between latent variables. However, applying both approaches in complex multilevel contexts, especially when involving multiple random effects, poses serious computational challenges under ML estimation, including slow run times and convergence failures due to high-dimensional integration.

Bayesian estimation offers a promising alternative to these limitations (Jedidi and Ansari, 2001; Preacher et al., 2016). Unlike ML estimation, Bayesian approaches directly sample from the posterior distribution, offering greater flexibility in handling complex, multilevel data structures (Keller and Enders, 2023; Yuan and MacKinnon, 2009). Bayesian approaches are particularly well-suited for models involving latent variables. Particularly, Bayesian latent interaction demonstrates improved convergence rates when adapted in a Bayesian context, allowing for more effective estimation of interactions between latent variables (Keller, 2024). Thus, Bayesian estimation facilitates the analysis of complex models, especially those involving latent moderated mediation within multilevel contexts.

This study compares the efficacy of Bayesian random coefficient prediction (BRCP) and Bayesian latent interaction (BINT) models in estimating moderated mediation effects in multilevel contexts. By analyzing empirical data from the 2019 Trends in International Mathematics and Science Study (TIMSS; Fishbein et al., 2021) dataset and simulated datasets, we evaluate the performance of these models in estimating latent moderated mediation effects. Specifically, we (1) compare the effectiveness of BRCP and BINT in estimating moderated mediation effects, (2) assess their performance across different simulation conditions, and (3) provide practical guidelines for researchers in selecting the appropriate method for their research questions and data structures. This study contributes in two ways: by expanding model specification to multilevel moderated mediation with multiple random slopes which are not feasible in ML, and by comparing two algebraically equivalent parameterizations—BRCP and BINT—under Bayesian estimation.

We structured the balance of the paper as follows. First, we briefly elaborate on the theoretical foundations of MSEM and latent moderation modeling, including the integration of between-level covariates in within-level models. Next, we provide an empirical analysis using TIMSS2019 data to compare the performance of BRCP and BINT models in estimating multilevel moderated mediation effects. Next, we discuss a simulation study to validate our findings, concluding with a discussion on the implications and recommendations for future research.

## Multilevel structural equation modeling

Multilevel structural equation modeling (MSEM) combines the strengths of multilevel modeling (MLM) and structural equation modeling (SEM), addressing nature of the sampling process in hierarchical data and measurement error in latent constructs (Stapleton, 2013). MLM is suited for analyzing data with nested structures, like students within schools, by accounting for the dependency among observations within clusters. On the other hand, SEM accounts for measurement error while modeling complex relationships involving observed and latent variables. By integrating these two methodologies, MSEM enables simultaneously addressing the latent constructs and nested data structures. To address sampling structure in nested data, researchers decompose the variance into between-group and within-group components. Equation 1 represents the decomposition.


$$\begin{array}{l} y_{ig}=y^{B}+y^{W}=\alpha +u_{g}+e_{ig} \end{array}$$ (1)

where subscription *i* means individual-level, *g* means group-level, and *y*_*ig*_ is the observed variable. *y*^*B*^ and *y*^*W*^ are the between- and within-group components, respectively. α represents the grand mean, *u*_*g*_ is the group-level residual, and *e*_*ig*_ is the individual-level residual. This decomposition allows for the separation of variance components (Σ) attributable to individual differences within groups (within-group variance) and differences between groups (between-group variance), such as Σ = Σ^*B*^+ Σ^*W*^.

Equation 2 formulates the measurement model for the decomposed latent variables using latent centering:


$$\begin{array}{l} y^{B}=\nu^{B}+\Lambda^{B}\eta_{g}^{B}+\theta_{g}^{B},\\ y^{W}=\Lambda^{W}\eta_{ig}^{W}+\;\theta_{ig}^{W} \end{array}$$ (2)

Here, Λ^*W*^ and Λ^*B*^ denote the factor-loading matrices for the within- and between-level indicators, respectively. ν^*B*^ is the vector of latent means at the between level. $\eta_{ig}^{w}$ represents factors that vary randomly across individuals within groups and $\eta_{g}^{b}$ represents factors that vary across groups. Further, $\theta_{ig}^{W}$ and $\theta_{g}^{B}$ are the residual variances for the within-group and between-group models captured in diagonal matrices Θ^*w*^ and Θ^*b*^, respectively (Kaplan, 2008). Incorporating this measurement model into the structural model leads to the reformulated model in Equation 3.


$$\begin{array}{l} y_{ig}=\nu^{B}+\Lambda^{W}\eta_{ig}^{W}+\Lambda^{B}\eta_{g}^{B}+\theta_{ig}^{W}+\theta_{g}^{B} \end{array}$$ (3)

With latent factor measurement structures, we express the structural model in MSEM as follows:


$$\begin{array}{l} \eta =\text{B}\eta +\Gamma \xi +\zeta \end{array}$$ (4)

where η is the vector of endogenous latent variables, ξ represents exogenous latent variables, B and Γ represent relationships among latent constructs, and ζ denotes latent residuals. This framework enables MSEM to model latent relationships and hierarchical dependencies simultaneously, making it a robust tool for understanding complex phenomena in the social and behavioral sciences.

### Multilevel mediation in MSEM

Building on the capabilities of MSEM, we can further explore mediation effects at the within-group and between-group levels. Mediation analysis is especially useful for understanding the mechanisms through which predictors influence outcomes, and MSEM extends this understanding to hierarchical data structures, which are common in psychology and education research (Bauer et al., 2006). These mediations, or indirect effects, help explain how an independent variable (X) affects a dependent variable (Y) through an intervening mediator (M). Within mediation modeling, the a-path represents the effect of a predictor (X) on the mediator (M), while the b-path represents the effect of the mediator (M) on the outcome (Y). The product of these two paths (ab) gives the indirect effect, and the direct effect of X on Y is the c′ path, with c = ab + c′ representing the total effect (Bollen, 1987).

In MSEM, researchers design mediation models with varying complexity depending on the measurement level of each variable, using structures like 1-1-1, 2-1-1, or 2-2-1 (Preacher et al., 2010). This study focuses on the 1-1-1 mediation model, where we measure the predictor, mediator, and outcome at the individual level (Level 1). This model provides a foundational understanding of mediation by examining individual-level relationships while also accounting for variance within clusters (individual level) and between clusters (group level).

In a 1-1-1 multilevel mediation model, the mediation process operates in the within-level (Level 1) and between-level (Level 2). The model examines how a Level 1 predictor (X) affects a mediator (M) at the individual level and how the mediator subsequently influences an outcome (Y). We represent this relationship by the a-path (effect of X on M) and the b-path (effect of M on Y). This model enables the analysis of relationships at the individual level while simultaneously modeling relationships at the group level, allowing for a comprehensive examination of mediation effects that arise at both levels within a single model.

One important aspect of multilevel mediation in MSEM is when researchers treat the a- and b-paths as random variables that can correlate. Unlike single-level mediation models, where the indirect effect (a × b) is the product of individual paths, in multilevel models, *E*(*a*)*E(*b)+σ_*ab*_ gives the expected value of the product *E*(*a*_*j*_*b*_*j*_), where σ_*ab*_ represents the population covariance between *a*_*j*_ and *b*_*j*_, accounting for the potential correlation between these effects (Goodman, 1960)^2^. A positive σ_*ab*_ means that higher-level units with larger *a*_*j*_ values are likely to also have larger *b*_*j*_ values. On the other hand, a negative σ_*ab*_ implies that units with higher *a*_*j*_ values are more likely to have smaller *b*_*j*_ values (Kenny et al., 2003).

### Cross-level moderation via random coefficient prediction

Building on the multilevel mediation framework discussed earlier, we now turn to cross-level moderation, specifically through the random coefficient prediction (RCP) approach. In multilevel mediation, researchers are curious about how within-cluster effects, such as the a, b, and c′ paths of mediation models, vary across clusters and how Level 2 covariates influence these relationships (Bauer et al., 2006). In other words, mediation effects are not fixed but can vary across groups, such as schools or organizations, depending on cluster-level characteristics.

To capture this variability, we adapted the RCP approach, which involves introducing random slopes for mediation paths to allow effects to vary between clusters. This type of cross-level moderation allows the Level 2 covariates to moderate the relationships within Level 1 (e.g., the effect of X on M and M on Y; Raudenbush and Bryk, 2002). The random slopes essentially become outcome variables that cluster-level moderators can predict, thus providing insight into how and why these within-group effects change across different contexts (Preacher et al., 2016). For example, the relationship between students' interactions with their mathematics (herein, “math”) teachers (X) and their interest in math (Y) might vary across different schools. By modeling this relationship using RCP, we treat the random slope as a latent variable that school-level characteristics, such as teacher–student ratio or available resources, can explain. This approach helps uncover why the strength of this relationship differs across schools and how contextual factors influence individual outcomes.

In MSEM, incorporating cross-level moderation with random slopes adds complexity, but it also deepens the understanding of variability across contexts. This approach provides a more comprehensive view of how individual-level relationships change across clusters, offering clearer insights into moderation within hierarchical data structures (Kaplan, 2008).

### Latent interaction modeling for cross-level moderation

Latent interaction modeling (LMS) offers an alternative approach to RCP for examining cross-level moderation within MSEM. While RCP estimates random slopes to capture how between-level covariates influence within-level coefficients, LMS takes a different route by directly modeling these interactions through latent interaction terms in the structural model, simplifying the specification of cross-level interaction. In RCP, cross-level moderation often involves Level 2 random slopes derived from Level 1 variables to examine how these slopes vary based on the level of Level 2 covariates. In contrast, LMS incorporates interaction terms directly between Level 1 and Level 2 variables as predictors, making it an efficient way to model interactions without relying on random slope estimation.

Preacher et al. (2016) proposed this latent interaction approach under maximum likelihood estimation (MLE) using latent moderated structural equations (Klein and Moosbrugger, 2000). By creating product terms involving latent variables from different levels, LMS captures cross-level interactions more directly. LMS uses latent product terms to model the interaction between a Level 1 predictor (e.g., student motivation) and a Level 2 covariate (e.g., school resources) rather than estimating how a slope varies across groups in response to a moderator. This approach allows researchers to estimate the influence of group-level moderators directly on within-level relationships, providing a straightforward pathway to understanding between-level moderation.

Thus, while RCP treats random slopes as outcomes influenced by moderators, LMS bypasses this step by modeling the moderation effect as a fixed interaction term between latent predictors. Both approaches offer distinct modeling strategies but ultimately address the same conceptual question.

### Equivalence of cross-level moderation in RCP and LMS models

Although the different specifications of cross-level moderation of RCP and LMS, these two approaches are mathematically equivalent in representing the moderation. To maintain conciseness, the simplest cross-level interaction is illustrated. In RCP, the random slope, β_*YX*_, of a level 1 predictor (*X*_*w*_) can be modeled as a function of a level 2 moderator (*Z*_*B*_):


$$\begin{array}{l} \text{ Level }1:\;Y_{W}=\beta_{YX}X_{W}+\beta_{YZ}Z_{W}+\varepsilon_{Y_{W}},\\ \text{ Level }2:\;Y_{B}=\;\gamma_{Y_{B}}+\gamma_{YX}X_{B}+\gamma_{YZ}Z_{B}+\varepsilon_{Y_{B}},\\ \;\beta_{YX}=\gamma_{\beta_{YX}}+\gamma_{\beta Z}Z_{B}+\varepsilon_{\beta_{YX}} \end{array}$$ (5)

Substituting random coefficient at level-2, β_*YX*_, into the level-1 model:


$$\begin{array}{l} \text{Level }1\;\left(\text{mixed}\right):\;Y_{W}=\;\gamma_{\beta_{YX}}X_{W}+\gamma_{\beta Z}X_{W}Z_{B}+\varepsilon_{\beta_{YX}}X_{W}\\ \;+\;\beta_{YZ}Z_{W}+\varepsilon_{Y_{W}},\\ \text{ Level }2:\;Y_{B}=\;\gamma_{Y_{B}}+\gamma_{YX}X_{B}+\gamma_{YZ}Z_{B}+\varepsilon_{Y_{B}}, \end{array}$$ (6)

where γ_β*Z*_*X*_*W*_*Z*_*B*_ represents the cross-level interaction, capturing the moderating effect of the level-2 variable *Z*_*B*_ on the relationship between *X*_*W*_ and *Y*_*W*_. In other words, the strength of the level-1 association varies depending on the level-2 context.

In contrast, the LMS approach models the cross-level moderation, β_*cross*_*X*_*W*_*Z*_*B*_, directly in the structural model:


$$\begin{array}{l} \text{Level }1:\;Y_{W}=\beta_{YX}X_{W}+\beta_{YZ}Z_{W}+\beta_{cross}X_{W}Z_{B}+\varepsilon_{Y_{W}},\\ \text{ Level }2:\;Y_{B}=\;\gamma_{Y_{B}}+\gamma_{YX}X_{B}+\gamma_{YZ}Z_{B}+\varepsilon_{Y_{B}},\\ \;\beta_{YX}=\gamma_{\beta_{YX}}+\varepsilon_{\beta_{YX}}. \end{array}$$ (7)

Substituting level-2 β_*YX*_, into the level-1 model, it yields:


$$\begin{array}{l} \text{Level }1\;\left(\text{mixed}\right):\;Y_{W}=\;\gamma_{\beta_{YX}}X_{W}+\varepsilon_{\beta_{YX}}X_{W}+\\ \;\beta_{YZ}Z_{W}+\beta_{cross}X_{W}Z_{B}+\varepsilon_{Y_{W}},\\ \text{ Level }2:\;Y_{B}=\;\gamma_{Y_{B}}+\gamma_{YX}X_{B}+\gamma_{YZ}Z_{B}+\varepsilon_{Y_{B}}, \end{array}$$ (8)

Comparing the mixed models in Equations 6 and 8 reveals that β_*cross*_ captures the same moderation effect as γ_β*Z*_ in the RCP. While the estimation strategies and model formulations differ, the moderation effect is equivalent across the two approaches.

### Estimation challenges: random coefficient prediction and latent moderated structural equations

Estimating cross-level moderation models in MSEM, such as RCP and LMS, poses significant challenges, particularly due to the complexities involved in estimating random slopes and latent interactions. These models often require full information, and ML estimation is typically necessary for models involving random slopes, relying on computational methods like the expectation-maximization (EM) algorithm, which can struggle with convergence and model identifiability (e.g., Holtmann et al., 2016). Bayesian estimation emerges as a promising alternative to address these challenges. Unlike ML, Bayesian methods use Markov Chain Monte Carlo (MCMC) to sample posterior distributions directly, allowing for more robust and flexible estimation. This study addresses convergence issues by employing Bayesian estimation, which offers a more direct modeling approach (Keller, 2024; Keller and Enders, 2023; Keller, 2022).

By incorporating product terms between random effects and covariates, Bayesian methods—like factored regression modeling in Blimp—allow for a more precise estimation of interaction effects. From an applied researcher's perspective, the factored regression approach for cross-level moderation is as straightforward as using standard moderated regression. The Bayesian framework also incorporates prior distributions, which aids in addressing computational limitations and provides more nuanced insights into model parameters, even for complex multilevel moderation models.

In Bayesian estimation, we define the full posterior distribution of model parameters given the observed data as:


$$\begin{array}{l} P\left(\Omega |Y\right)\propto p\left(Y|\Omega \right)p\left(\Omega \right) \end{array}$$ (9)

where Ω represents the model parameters in Equation 9, *p*(*Y*|Ω) is the likelihood function of the multilevel SEM, and *p*(Ω) denotes the prior distributions for those parameters. We utilize MCMC sampling via the software Blimp (Keller and Enders, 2021) to approximate these posterior distributions, employing techniques like Gibbs sampling (Gelman et al., 1995; Jackman, 2009).

One notable strength of Bayesian estimation, beyond its computational efficiency, is that it provides parameter estimates along with credible intervals by constructing the entire posterior distribution. This approach offers several advantages over traditional frequentist methods, especially when testing mediation effects (Yuan and MacKinnon, 2009). Unlike traditional methods, which often struggle with the asymmetrical distribution of indirect effect estimates, Bayesian mediation analysis constructs credible intervals directly from the posterior distribution without requiring large sample approximations or normality assumptions (Kaplan and Depaoli, 2012). During MCMC sampling, the indirect effects at both the within-level and between-level models are drawn to form posterior distributions, from which credible intervals can be constructed. This approach is especially useful because the distribution of the product of two parameters, such as mediation effects, is often unknown and can be asymmetrical. Bayesian estimation avoids restrictive distributional assumptions, such as normality, that traditional methods may require. Through repeated sampling, Bayesian methods allow researchers to assess the exact shape of the posterior distribution, providing a more accurate picture of mediation effects in the complex multilevel models.

The Bayesian methods enable this study to demonstrate that researchers can effectively estimate BRCP and BINT in the MSEM framework, overcoming the limitations of ML estimation and providing robust, reliable parameter estimates along with straightforward interpretation and evaluation of indirect effects. This investigation will help clarify the suitability and performance of BRCP and BINT for multilevel moderated mediation models, contributing to best practices for researchers dealing with complex hierarchical data.

This study leverages the advantages of Bayesian MSEM to estimate and compare BRCP and BINT approaches for cross-level moderation. Specifically, we (1) apply MSEM to empirical data from TIMSS to evaluate cross-level moderation effects using the BRCP and BINT approaches with Bayesian estimation and (2) verify findings through a simulation study to determine whether observations from the empirical data are consistent under controlled conditions.

## Real data example

We derived the data for this study from the 2019 Trends in International Mathematics and Science Study (TIMSS; Fishbein et al., 2021) to compare the effectiveness of the Bayesian random coefficient prediction (BRCP) and Bayesian latent interaction (BINT) models in detecting mediated moderation effects. Specifically, this study uses the TIMSS 2019 eighth-grade data representing the U.S. population, encompassing 273 schools and 8,698 students.

TIMSS is a comprehensive assessment that helps participating countries gain valuable insights to improve student learning in mathematics and science (Mullis et al., 2005). The 2019 assessment includes achievement tests in mathematics and science alongside student background information. Given that the TIMSS survey collected data across schools, they are well-suited for applying MSEM with mediated moderation effects. Table 1 shows descriptive statistics of variables used in the analysis.

**Table 1:** Descriptive statistics for math teacher relationships, math class environments, math preference, and relatedness.

**Variable**	**Mean**	**SD**	**Skewness**	**Kurtosis**	**ICC**	**Missing (%)**
MTR1	3.573	0.682	−1.704	2.822	0.065	5.415
MTR2	3.258	0.897	−1.019	0.108	0.143	5.553
MTR3	3.278	0.894	−1.041	0.123	0.121	5.990
MTR4	3.379	0.878	−1.313	0.789	0.165	5.817
MTR5	3.388	0.857	−1.313	0.865	0.117	5.955
MTR6	3.317	0.857	−1.124	0.461	0.096	6.047
MTR7	3.417	0.863	−1.425	1.143	0.110	5.852
MCE1	2.432	0.959	−0.119	−0.996	0.138	6.024
MCE2	2.382	1.012	−0.032	−1.160	0.144	6.197
MCE3	3.053	0.980	−0.721	−0.561	0.165	7.048
MCE4	2.749	1.035	−0.413	−0.976	0.173	6.438
MCE5	2.625	1.057	−0.288	−1.131	0.162	6.565
MCE6	2.929	1.077	−0.617	−0.915	0.196	6.335
MP1	2.825	1.002	−0.497	−0.803	0.063	5.128
MP2	2.542	1.074	−0.028	−1.257	0.048	5.622
MP3	2.391	1.017	0.174	−1.075	0.044	6.956
MP4	2.848	0.962	−0.448	−0.756	0.051	6.300
MP5	2.721	1.058	−0.348	−1.092	0.061	7.519
MP6	2.334	0.985	0.192	−0.991	0.042	5.702
MP7	2.554	1.043	−0.117	−1.164	0.046	5.886
MP8	2.468	1.047	0.009	−1.191	0.064	5.932
MP9	2.481	1.181	0.005	−1.499	0.071	5.610
Relatedness	3.018	0.709	−0.675	−0.110	0.080	0.044

MTR, mathematics (“math”) teacher relationships (i.e., student–teacher relationships); MCE, math class environments; MP, math preference.

### Measure

The variables analyzed are “math preference (MP; BSBM16),” “math class environment (MCE; BSBM18),” and “relationship with math teachers (MTR; BSBM17),” measured by nine, six, and seven indicators, respectively. We measured the cross-level moderator “relatedness” (REL; BSBG13) with five items. We averaged the indicators of relatedness to create a composite score, which we used as a moderator at the between-level. In this context, the between-level relatedness moderates the indirect effect at the within-level of the math class environment on math preference via the relationship with math teachers. For illustrative purposes, the models presented here include a limited number of covariates. In practice, researchers could incorporate additional covariates at the between- and within-levels.

### Analytical strategy

**Figure 1:**
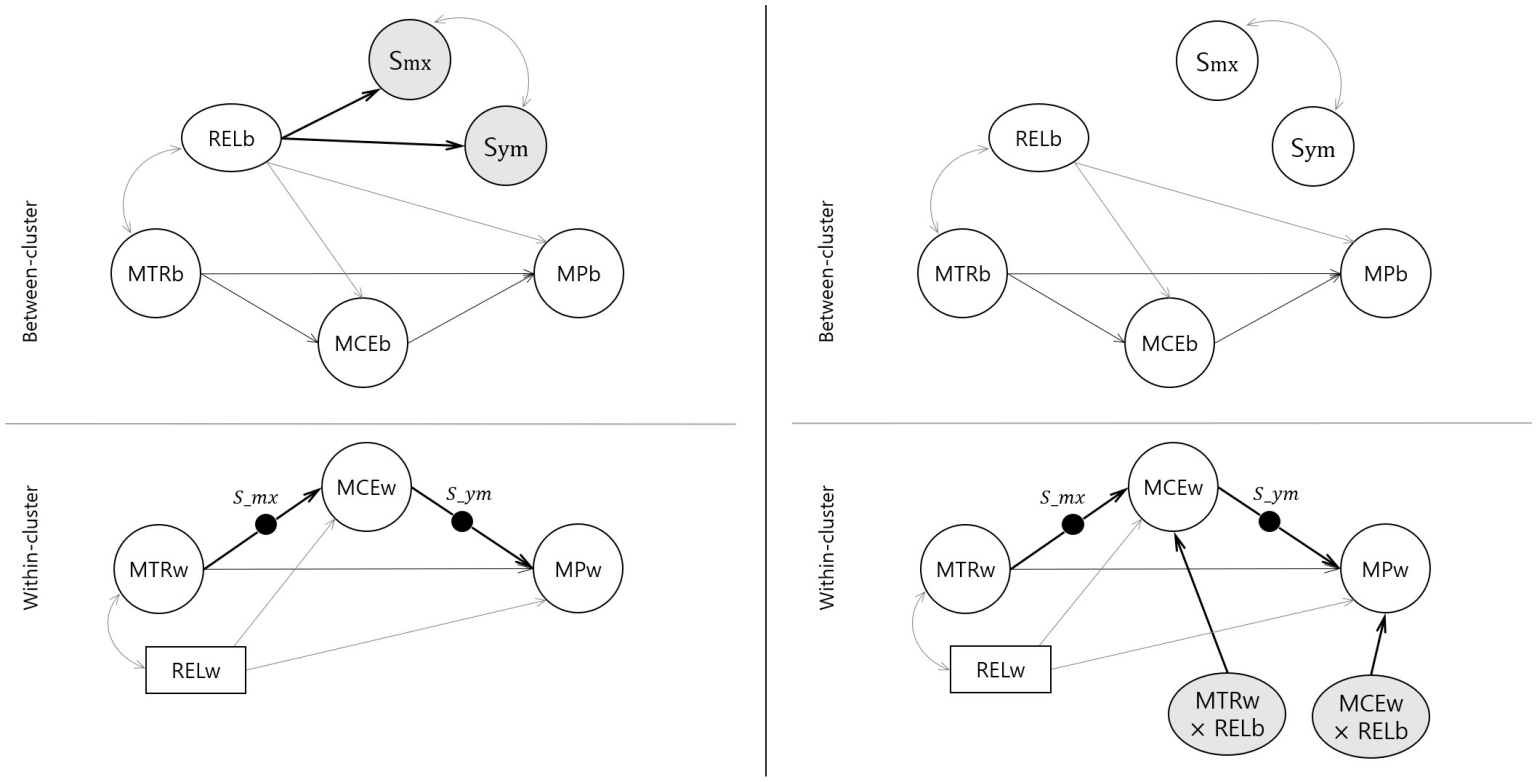
Bayesian random coefficient prediction (left) and Bayesian latent interaction (right) models with cross-level moderation for relationships among math class environments, math teacher relationships, and math preferences with relatedness as a covariate. Subscription b means between-level, sub w means within-level and upper scale S means random slope originated from within level. MTR, math teacher relationship; MCE, math class environment; MP, math preference; REL, school relatedness. *S*_*mx*_, random slope from MTRw to MCEw; *S*_*ym*_, random slope from MCEw to MPw.

We measured three latent variables at the student level—math preference (MP), math class environment (MCE), and math teacher relationship (MTR)—specifying a 1-1-1 mediation model. We also measured relatedness (REL), a moderator, at the student level. We originally measured relatedness with five indicators, which we averaged and treated as an observed variable to simplify the model. Figure 1 illustrates each model of the BRCP and BINT.

In the BRCP model, we specified two random slopes for the within-level mediation paths (a path and b path), treating both slopes as latent variables at the between-level. In the BINT model, we included interaction terms of within-level MTR and between-level REL and within-level MCE and between-level REL. We specified each path of the indirect effect as random coefficients and estimated the covariance between two random effects. To ensure model identification, we fixed the first factor loadings to “1” at both levels and estimated the intercepts of the Level 2 indicators. Additionally, we constrained the means of the latent factors to zero.

We used the Blimp program to conduct analysis (Keller and Enders, 2021) and the Gibbs sampler for Bayesian estimation. We applied the following prior distributions: normal distributions for regression coefficients and factor loadings, inverse-gamma distributions for residual variances, and inverse Wishart distribution for covariances and random effect residuals. We chose these distributions as they are the program defaults and conjugate priors, ensuring minimal influence on the posterior distribution (Kaplan and Depaoli, 2012).

We ran four Markov Chain Monte Carlo (MCMC) chains, each with 40,000 iterations, using the first 30,000 iterations as the warm-up phase^3^. We derived the MCMC estimates for each parameter from the medians of their posterior distributions. Estimated coefficients were significant when the 95% credible interval, defined by the 2.5th and 97.5th percentiles, did not include zero. The data and code for analysis with Bimp are available at [OSF: https://osf.io/3as45/?view_only=3f3173a69844400394eab43ae738c017]. Both models required approximately 40 min to complete the Bayesian estimation on a personal computer (AMD Ryzen 7 5700X, 3.85 GHz base clock), indicating identical computation times.

## Real data analysis results

### Model fit

Prior to conducting the main analysis, we performed a multilevel confirmatory factor analysis (CFA) for three factors: math preference (MP), math class environment (MCE), and math teacher relations (MTR). The results showed reasonable model fits and intraclass correlation coefficients (ICCs) for all three factors.

The ICCs ranged from 0.04 to 0.07 for MP across nine items, indicating that 4% to 7% of the variance in MP is attributable to differences between schools. The model fit indices demonstrated a reasonable fit with a comparative fit index (CFI) of 0.95, a Tucker–Lewis Index (TLI) of 0.93, and a root mean square error of approximation (RMSEA) of 0.075. Next, MCE demonstrated higher ICCs, ranging from 0.13 to 0.19 across six items, suggesting that 13% to 19% of the variance in MCE is due to between-cluster differences. The model fit indices were also acceptable (CFI = 0.96, TLI = 0.94, RMSEA = 0.07). Lastly, MTR showed ICCs between 0.07 and 0.16 across seven items, indicating that 7% to 16% of the variance in MTR is attributable to between-cluster differences. The model fit indices indicated a good fit (CFI = 0.96, TLI = 0.94, RMSEA = 0.07).

These results indicate acceptable model fits for all three factors, with CFI and TLI values approaching or exceeding 0.90 and RMSEA values below 0.08. The ICCs, particularly for MCE and MTR, suggest substantial between-cluster variability, supporting the use of multilevel modeling. Given these reasonable fit indices and the magnitude of the ICCs, we proceeded with multilevel structural equation modeling (MSEM) to examine the mediation and moderation effects in our hypothesized model.

### Model comparisons

Table 2 presents the estimates of multilevel mediation with cross-level interactions for both methods; Table 3 shows the results of mediation and moderated mediation effects for BRCP and BINT models. Overall, results revealed similarities between the BRCP and BINT models, with only negligible differences in parameter estimates, consistent with findings from Preacher et al. (2016), which relied on maximum likelihood (ML) estimation.

**Table 2:** Results of Bayesian random coefficient prediction (BRCP) and Bayesian latent interaction (BINT) models with school-level relatedness as the cross-level moderator.

**Path**	**Label**	**BRCP**	**BINT**
**Within-level**			
*MTR*_*w*_ ➔ *MCE*_*w*_	*a*_*w*_ (random)	**0.155** (0.094, 0.217)	**0.156** (0.093, 0.218)
*MCE*_*w*_ ➔ *MP*_*w*_	*b*_*w*_ (random)	**0.036** (0.003, 0.070)	**0.036** (0.002, 0.069)
*MTR*_*w*_ ➔ *MP*_*w*_	$c_{w}^{\prime }$	**0.703** (0.647, 0.761)	**0.704** (0.648, 0.762)
*REL*_*w*_ ➔ *MP*_*w*_		**0.348** (0.320, 0.377)	**0.348** (0.320, 0.377)
*REL*_*w*_ ➔ *MCE*_*w*_		**0.173** (0.150, 0.200)	**0.173** (0.148, 0.198)
**Between-level**			
*MTR*_*b*_ ➔ *MCE*_*b*_	*a*_*b*_	**0.774** (0.477, 1.100)	**0.770** (0.478, 1.088)
*MCE*_*b*_ ➔ *MP*_*b*_	*b*_*b*_	**0.202** (0.057, 0.347)	**0.201** (0.062, 0.348)
*MTR*_*b*_ ➔ *MP*_*b*_	$c_{b}^{\prime }$	**0.748** (0.454, 1.064)	**0.745** (0.455, 1.05)
*REL*_*b*_ ➔ *MP*_*b*_		**0.660** (0.473, 0.852)	**0.664** (0.476, 0.860)
*REL*_*b*_ ➔ *MCE*_*b*_		−0.092 (−0.290, 0.10)	−0.091 (−0.291, 0.106)
**Random slope**			
Variances	*a*_*w*_ (in btw)	**0.089** (0.059, 0.130)	**0.089** (0.059, 0.130)
	*b*_*w*_ (in btw)	**0.017** (0.006, 0.031)	**0.017** (0.007, 0.030)
Covariance		0.009 (−0.065, 0.025)	0.008 (−0.058, 0.022)
**Cross-level interactions**			
*REL*_*b*_➔ *a*_*w*_ (or *REL*_*b*_×*MTR*_*w*_ ➔ *MCE*_*w*_)		**0.291** (0.029, 0.563)	**0.295** (0.026, 0.568)
*REL*_*b*_ ➔ *b*_*w*_ (or *REL*_*b*_*xMCE*_*w*_ ➔ *MP*_*w*_)		**0.228** (0.072, 0.381)	**0.227** (0.072, 0.383)

The numbers in parentheses are 95% credible intervals (Crd.I). The bold values indicate cases where the Crd.I does not include zero. MP, math preference; MTR, math teacher relationship; MCE, math class environment; REL, school relatedness. The a path and b path of the BRCP within-levels are the intercepts of random slopes. btw, between-level.

**Table 3:** Results of mediation and cross-level moderated mediation by ±1 SD of school-level relatedness.

	**BRCP**	**BINT**
**Mediation**		
Within	0.015 (−0.002, 0.032)	0.014 (−0.001, 0.029)
Between	0.152 (0.041, 0.302)	0.151 (0.044, 0.299)
**Moderated mediation**		
+1 SD *REL*_*b*_	**0.029** (0.009, 0.053)	**0.028** (0.009, 0.050)
−1 SD *REL*_*b*_	0.008 (−0.009, 0.024)	0.007 (−0.008, 0.022)

The numbers in parentheses are 95% credible intervals (Crd.I). The bolded numbers indicate cases where the Crd.I does not include zero. SD, standard deviations of relatedness; BRCP, Bayesian random coefficient prediction; BINT, Bayesian latent interaction; Estimated SD of *REL*_*b*_ is 0.237.

The path coefficients of the within-school level for the BRCP and BINT models were as follows. The effect of math class environment on math teacher relationship (a path) showed a coefficient of 0.16, while the effect of math preference on math class environment (b path) was 0.04. Additionally, the effect of math preference on math teacher relationship (c′ path) was 0.70. These indicate that better student–teacher relationships (MTR) were associated with a less favorable math class environment (MCE), a more favorable MCE correlated with better math preference (MP), and MTR had a strong direct positive effect on MP at the student level.

The results at the between-school level were as follows. The effect of math class environment on math teacher relationship (a path) showed a coefficient of 0.77, while the effect of math preference on math class environment (b path) was 0.20. Additionally, the direct effect of math preference on math teacher relationship (c′ path) was 0.75. These results largely mirrored the direct path results of within-school effects, except for one key difference: in the case of, the between-schools relationship of MP on MCE was larger than the within-school relationships.

In terms of the random slopes, both models reported the variances of *a*_*w*_ and *b*_*w*_ as 0.09 and 0.02, respectively, which suggests the path of MCE on MTR and MP on MCE from the student level varies across schools. The covariance between two random slopes (*a*_*w*_ and *b*_*w*_) was below 0.01, and not statistically significant, as the 95% credible interval included zero. For the cross-level interaction results, school-level relatedness (*REL*_*b*_) accounted for this variability, amplifying these associations by 0.30 and 0.23, respectively, in both models.

The moderated mediation results in Table 3 were comparable across the two models, indicating that both models produced nearly identical outcomes. When relatedness was at a high level (+1 SD), the within-level mediation effects strengthened by 0.03 in both models, and the credible interval excluded zero, indicating statistical significance. This suggests that in schools with higher relatedness, the indirect effect from MCE to MP via MTR becomes stronger, implying that a supportive school climate can enhance the positive chain of associations among these variables. In contrast, when relatedness was at a low level (−1 SD), the within-school mediation effects showed a small coefficient of 0.01, and the credible interval included zero, indicating a lack of statistical significance. While this effect is not statistically reliable, its direction was consistent with the high-relatedness condition, hinting that even in less supportive schools, the mediation pathway may still operate, albeit weakly. Taken together, the variation in within-level mediation effects appears contingent on the level of school-level relatedness, providing partial but not definitive evidence of a cross-level moderated mediation.

To gauge the similarity in parameter estimates between the BRCP and BINT models, we computed both the probability-based effect size and the posterior overlap coefficient with the posterior distributions of key model parameters (i.e., within-school and between-school structural paths, random slope variances, mediation effects, and moderated mediation effects; Ruscio, 2008; Pastore and Calcagnì, 2019). As a general guideline, an effect size below 0.2 and a posterior overlap coefficient above 0.9 indicate negligible differences between posterior distributions. Consistent with this threshold, the observed effect sizes across all parameters were close to zero, and all posterior overlap coefficients exceeded 0.9. These results provide strong evidence that BRCP and BINT yield nearly identical posterior distributions across all focal parameters, supporting the conclusion that the two modeling approaches produce substantively equivalent results. Full results, including posterior comparisons for all parameters, are presented in the Online Supplementary materials (https://osf.io/3as45/?view_only=3f3173a69844400394eab43ae738c017).

Thus, we run a simulation study to explore these similarities under different conditions, including varying sample sizes, random slope variances, and effect sizes. This additional research will provide insights into which model might be more appropriate in specific contexts, thereby offering researchers clearer guidance for detecting moderated mediation in a multilevel structural equation model (SEM) framework.

## Simulation study

We conducted a small Monte Carlo simulation to assess the performance of Bayesian random coefficient predictor (BRCP) and Bayesian interaction (BINT) models in detecting cross-level moderation effects in mediated multilevel structural equation modeling (MSEM). The simulation incorporated various conditions, including scenarios where the between-level covariate moderates the random slopes, similar to the setup used in the empirical study discussed earlier. We used Blimp to estimate the Bayesian BRCP and BINT models Blimp (Keller and Enders, 2021). The study limited itself to 100 replications per condition due to the computational demands of Bayesian estimation.

### Design and conditions

The simulation design manipulated four key conditions based on the MSEM model from the previous empirical study, structuring three latent factors according to 1-1-1 mediation models and using a covariate as a cross-level moderator. To simplify notation, we referred to the three factors—MTR (math teacher relationship), MCE (math class environment), and MP (math preference)—as FX, FM, and FY, with the suffixes “w” and “b” denoting within- and between-levels, respectively. We labeled the covariate, school relatedness, Z.

The first condition varied the sample size. We fixed the Level 1 sample size at 30, while the number of Level 2 units was 20 or 100, resulting in total sample sizes of 1,000 (small sample) and 5,000 (large sample). The second condition manipulated the variance of the random slopes, set at 0 (no variance) or 0.50 (moderate variance). The final condition varied the magnitude of the moderation effect, set at 0 (no moderation) or 0.30 (moderate moderation).

We fixed several parameters based on empirical findings. Each factor had six indicators, which aligned with average values observed in real-world studies; we tested all indicators as continuous variables. Factor reliability, which influences factor loadings and residuals, was set at 0.60. We fixed path coefficients to reflect typical values: the a path (FX➔FM) was 0.4, the b path (FM➔FY) was 0.50, and the c′ path (FX➔FY) was −0.20, resulting in an overall mediation effect of 0.2 in the within- and between-cluster levels. We set the variances of the latent factors to 1, and the observed covariate Z had a variance of 0.50 at both levels, making its total variance 1 (standardized). At the within-cluster level, Z's effect on FMw was −0.10, and its effect on FYb at the between-cluster level was 0.10. Conversely, at the between-cluster level, Z's effect on FMb was −0.20, and its effect on FYb was 0.20. Additionally, the intraclass correlation coefficient (ICC) was set at 0.20, reflecting typical values observed in empirical data, capturing different levels of between-cluster variation.

### Analysis procedures

We fitted the BRCP and BINT models using the Gibbs algorithm for Bayesian estimation. For the prior settings, we used the same relatively non-informative conjugate priors as in the empirical study. To estimate the posterior distributions of the model parameters, we ran two Markov Chain Monte Carlo (MCMC) chains, each with 30,000 iterations. We used the additional 20,000 iterations as the burn-in phase to ensure that the chains had reached their stationary distributions. We applied the Gelman–Rubin convergence diagnostic (also referred to as R-hat or Potential Scale Reduction, PSR; Gelman and Rubin, 1992) to evaluate the convergence of the MCMC chains. This study considered any parameter with an R-hat value greater than 1.1 indicative of non-convergence. The final MCMC estimates came from the posterior distributions of the model parameters after ensuring convergence.

To ensure model identification, we fixed all of the first factor loadings for the three factors to one in the Level 1 and Level 2 models while freely estimating the variances of the latent factors. We constrained the latent means to zero while freely estimating the indicator intercepts at Level 2. We considered parameters statistically significant if their credible intervals did not include zero. For cross-level moderation detection, we identified moderation effects when the credible intervals, defined by the 2.5th and 97.5th percentiles of the posterior distributions, excluded zero. We used the R statistical software (R Core Team, 2024) to generate simulated data based on the real data example, which featured a moderated mediation model with three latent factors and one observed variable.

### Simulation outcome

This study assessed power and Type I error rates to compare the performance of BRCP and BINT in detecting moderated effects. We identified cross-level moderation effects when the credible interval of the related parameter excluded zero for both models, with BINT reflecting moderation effects and BRCP affecting the random slope. We calculated Type I error rates as the proportion of false positives in detecting moderation effects across replications, using a nominal rate set at 0.05. We determined power as the proportion of correctly identified moderation effects, considering rates above 0.8 as excellent and those between 0.70 and 0.80 as moderate (Cohen, 1992).

To evaluate the accuracy of the moderation effect and moderated mediation estimates, we analyzed average relative bias (RB). The difference between estimated and true parameter values divided by true parameters indicates the percentage of estimation errors. Acceptable RB is lower than 0.10, with RB close to zero representing better accuracy (Muthén and Muthén, 2002). We compared the RB of cross-level moderation and moderated mediation estimates obtained using the two approaches across all the simulation conditions.

### Simulation study results

#### Non-convergence

Two approaches resulted in good convergence rates of less than 3%. Despite the higher non-convergence rates in certain models, we did not observe extreme $\hat{R}$ values (e.g., exceeding 1.50 or 2). To mitigate non-convergence, we applied additional iterations to replications with PSR values exceeding 1.1, assessing whether more iterations would achieve convergence. After extending to 100,000 iterations, most replications reached convergence with PSR values below 1.1. Consequently, we retained replications with PSR under 1.1 or conducted further replications to ensure we had 100 converged replications per condition.

#### Accuracy of cross-level moderation

**Figure 2:**
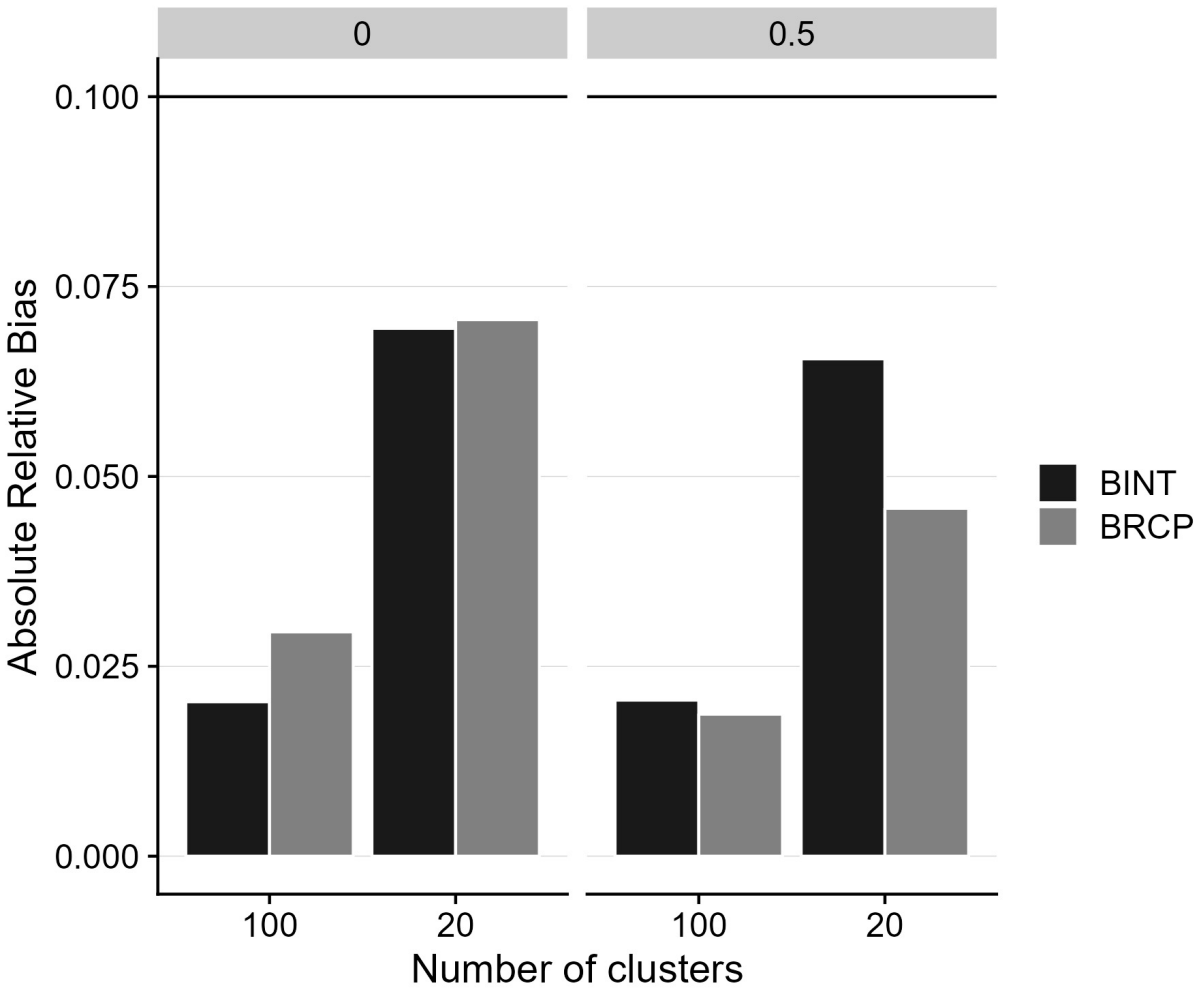
Absolute relative bias of cross-level moderation effects for Bayesian random coefficient prediction (BRCP) and Bayesian latent interaction (BINT) models. The top labels indicate the size of random slope variances. Calculations of relative bias are only for a moderation effect size of 0.3.

Figure 2 presents the relative bias of cross-level moderation effects for both estimation approaches (the 1-1-1 mediation model with two random slopes) under various conditions. To facilitate a clearer comparison of estimation accuracy, we averaged the absolute relative bias across the two moderation effects. Overall, both methods demonstrated relative bias within the acceptable threshold of 0.10 for most conditions. However, we observed an increase in bias when the cluster size was 20 compared to 100. Additionally, BINT exhibited slightly higher bias for cross-level moderation estimates, though all estimates remained within the acceptable 0.10 cutoff.

#### Type I errors and power of cross-level moderation

**Figure 3:**
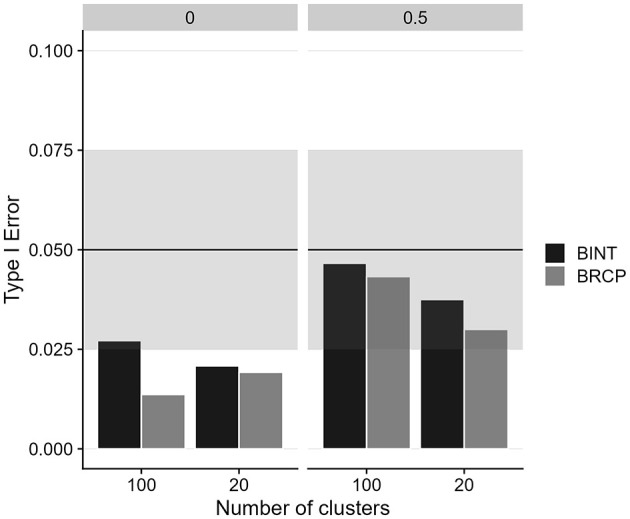
Type I error of cross-level moderation effects for Bayesian random coefficient prediction (BRCP) and Bayesian latent interaction (BINT) models. The top labels indicate the size of random slope variances.

**Figure 4:**
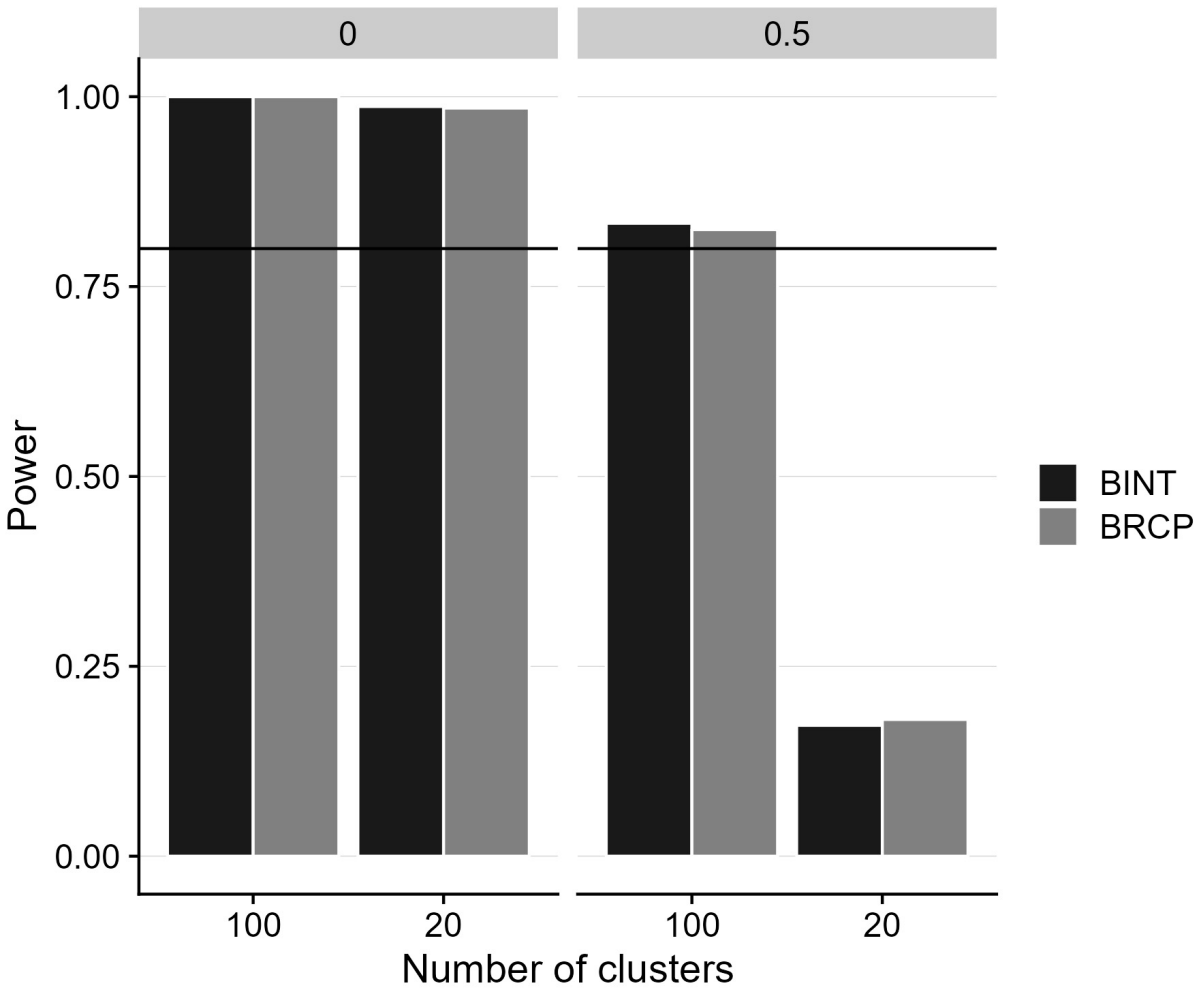
Power of cross-level moderation effects for Bayesian random coefficient prediction (BRCP) and Bayesian latent interaction (BINT) models. The top labels indicate the size of random slope variances.

Figures 3, 4 illustrate the power and Type I error rates for the BRCP and BINT methods in estimating cross-level moderation effects, focusing on the impact of sample size and random slope variance. Both approaches generally demonstrate similar proficiency in maintaining acceptable error rates across the simulated conditions. Type I errors are well-controlled for both methods, remaining below 0.05, indicating that neither BRCP nor BINT falsely detected cross-level moderation effects when none were present.

In terms of power, BINT and BRCP methods show comparable performance in detecting cross-level moderation effects, with power levels exceeding the 0.80 threshold, except when the cluster size was 20. In this smaller sample condition, detection power dropped to below 0.25, indicating low power to detect cross-level moderation. This result suggests the necessity of having a larger Level 2 sample size for reliable detection of cross-level moderation in multilevel structural equation modeling (SEM). With sufficient sample size, BRCP and BINT offer advantages regarding precise estimation of cross-level moderation effects, robustness in controlling Type I error rates and maintaining power in multilevel SEM.

#### Accuracy of moderated mediation effects

**Figure 5:**
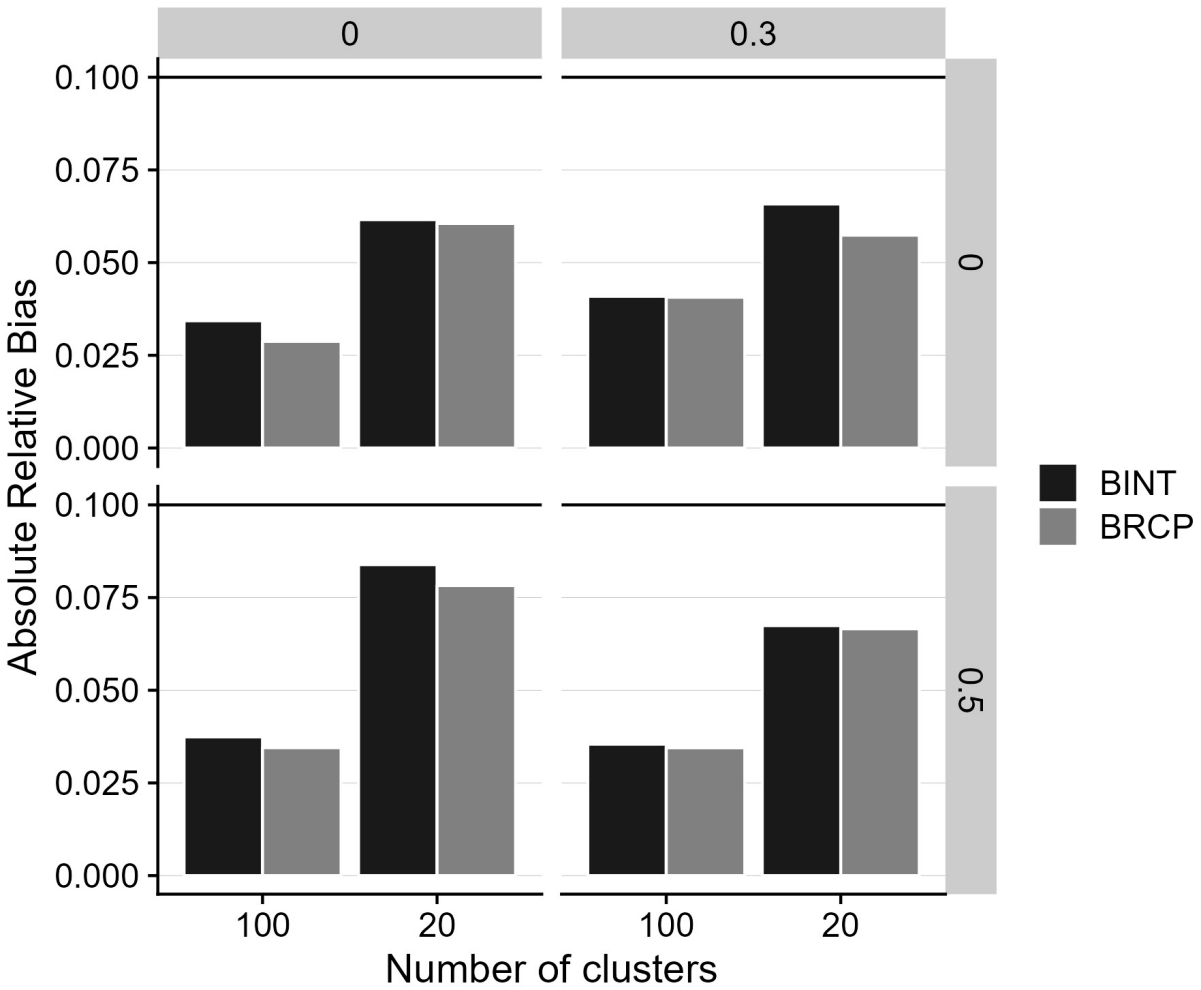
Absolute relative bias of moderated mediation effects for Bayesian random coefficient prediction (BRCP) and Bayesian latent interaction (BINT) Models. The first top labels indicate the size of moderation effects; the labels on the right indicate the size of random slope variances.

Figure 5 displays the relative bias of moderated mediation effects for both estimation approaches across various simulation conditions. Overall, the BRCP and BINT methods maintained relative bias within the commonly accepted threshold of 0.10. Bias was notably higher when the number of Level 2 units (cluster size) was 20. However, even with this smaller sample size, both models demonstrated consistent accuracy across conditions, keeping relative bias within the acceptable cutoff despite a slight increase in bias under the 20 cluster size condition.

## Discussion and limitations

The findings from both the empirical and simulation studies contribute significantly to our understanding of Bayesian methods for estimating moderated mediation effects in multilevel contexts. The results indicate that the Bayesian random coefficient prediction (BRCP) and Bayesian latent interaction (BINT) models produce highly similar parameter estimates, with only negligible differences, aligning with earlier research based on maximum likelihood estimation (MLE; Preacher et al., 2016). This consistency underscores the robustness of Bayesian estimation for analyzing moderated mediation models in hierarchical data structures. Below, we explore the implications, limitations, and potential avenues for future research arising from these findings.

The empirical results reveal that the BRCP and BINT models provide consistent estimates of within- and between-level relationships. The relationships between student–teacher relationships (MTR), math class environment (MCE), and math preference (MP) were largely similar across both models at the within- and between-school levels. This similarity suggests that researchers can expect comparable results regardless of whether they use BRCP or BINT when analyzing moderated mediation in multilevel models. The within-school relationship between school relatedness and class environment was positive (0.17), whereas the between-school relationship was negative (−0.09). Although the latter did not reach statistical significance, the difference in direction across levels may reflect school-level contextual factors that shape this association. For instance, structural constraints, such as limited resources, inefficient policies, or rigid administrative practices, may weaken the overall impact of school relatedness on the classroom environment, even when individual teachers are perceived as supportive. Additionally, unobserved school-level characteristics, such as average socioeconomic status by the school location, teacher's leadership style, may act as confounders, potentially obscuring the true between-school association.

The simulation study further validates the empirical findings, demonstrating that the BRCP and BINT models maintain acceptable levels of relative bias, even under challenging conditions such as small cluster sizes. Both methods produced generally similar results. The most notable difference, shown in Figure 2, was observed for the bias in cross-level moderation: when the level-2 sample size was small and there was variance among random slopes, BINT exhibited a larger bias than BRCP. Although the relative bias values for both BINT and BRCP were below 0.10—indicating both approaches are sufficiently accurate for most practical research settings—the results imply that under the harsh condition, BRCP may be preferable to BINT. Overall, the approach of incorporating latent interaction terms directly into the structural model appears to be more computationally demanding than the random slope approach. For instance, when estimating a latent moderated structural equation model including two cross-level interaction terms using ML in Mplus, the analysis failed to complete even after several days of continuous processing. Given the excessive computation time, the process was manually terminated. Although Bayesian estimation markedly improved convergence, the traditional slope-as-outcome method is presumed to be less computationally burdensome than the direct inclusion of cross-level interaction terms within the multilevel SEM. Furthermore, the well-controlled Type I error rates and comparable power levels of both models indicate their proficiency in accurately detecting cross-level moderation effects under a variety of conditions. However, both models struggled with power when the cluster size was as small as 20, emphasizing the importance of a sufficiently large sample size at Level 2 to achieve reliable estimates.

These findings have several important implications for researchers utilizing multilevel moderated mediation models. First, given the similarities between BRCP and BINT regarding accuracy and convergence, the choice between these two methods may depend more on specific research preferences or computational considerations rather than expected differences in results. The slight increase in bias for the BINT model under small cluster sizes suggests that researchers working with very small samples might prefer the BRCP model, although both approaches performed adequately within the commonly accepted thresholds.

Additionally, the strong performance of both models in controlling Type I errors and maintaining power reinforces their utility in complex multilevel analyses involving latent variables. Given the difficulty of estimating cross-level interactions with traditional multilevel models (i.e., MLE), the flexibility and robustness offered by Bayesian approaches provide a promising alternative. This situation is particularly relevant in applied fields such as education and psychology, where hierarchical data structures are common, there is significant interest in moderated mediation analysis, and the application of MLE poses considerable challenges.

Despite the strengths of the current study, some limitations warrant consideration. One is the relatively narrow range of conditions explored in the simulation. Although we varied sample sizes, random slope variances, and effect sizes, future research could explore additional factors, such as varying degrees of measurement error, the inclusion of more complex moderator structures, or different types of latent variable distributions. Such expansions would provide a more comprehensive understanding of the robustness of BRCP and BINT approaches across diverse research scenarios.

Although the inclusion of latent moderators is methodologically desirable, the current study focused on comparing model specifications for multilevel moderated mediation with multiple random slopes. Our aim was to evaluate the estimation performance and application of two distinct way—BRCP and BINT—incorporating Bayesian estimation. To maintain model comparability and avoid convergence issues with measurement-related complexities, we opted to model the moderator as a composite score. Introducing a latent moderator enables fuller utilization of SEM's strengths, namely, accounting for measurement error and modeling latent constructs, and stands as a promising avenue for future research.

Moreover, the observed decrease in power at small cluster sizes points to a critical consideration for researchers: the necessity of adequate Level 2 sample sizes in multilevel structural equation modeling analyses involving cross-level interactions. Future studies should investigate strategies to improve estimation accuracy and power in these conditions, potentially through alternative priors or hierarchical shrinkage techniques that could stabilize estimates when Level 2 units are limited.

In conclusion, this study provides valuable insights into the efficacy of BRCP and BINT models for detecting moderated mediation effects in multilevel contexts. The findings suggest that both models are reliable tools for researchers dealing with latent moderated mediation, particularly when sample sizes are sufficient. These insights can guide researchers in selecting appropriate modeling strategies based on their specific data conditions and research questions. Further exploration of these methods under varying and more complex conditions will enhance our ability to capture and interpret the nuanced relationships inherent in multilevel data structures.

## Data Availability

Publicly available datasets were analyzed in this study. This data can be found at: https://timssandpirls.bc.edu/databases-landing.html.

## Footnotes

1 In this study, however, we employ Bayesian estimation using factored regression methods for interaction-based models. Unless otherwise specified, we will interchangeably refer to LMS as the Bayesian Interaction Model (BINT).

2 By the definition of covariance, σ_*ab*_ = *E*(*ab*)−*E*(*a*)*E*(*b*). This covariance term is only relevant when both mediation paths (a and b) are treated as random effects. If only one path is random—for example, if a-path is random and b-path is fixed—then E(ab) simplifies to E(a) × b.

3 TIMSS provides sampling weights to support population-level inferences by adjusting for the complex sampling design, including school- and student-level selection probabilities and nonresponse. However, due to convergence issues when applying weights in Blimp and because the goal of this study is to evaluate estimation methods for mediation and moderation in multilevel models rather than to generalize empirical findings to the broader population, sampling weights were not applied in the current analyses. As such, the empirical results should be interpreted as illustrative of model behavior rather than as population-representative estimates. For further guidance on incorporating sampling weights in multilevel models and TIMSS data, see Asparouhov (2006) and the TIMSS 2019 User Guide (Fishbein et al., 2021).
